# Backbone and nearly complete side-chain chemical shift assignments reveal the human uncharacterized protein CXorf51A as intrinsically disordered

**DOI:** 10.1007/s12104-021-10043-6

**Published:** 2021-08-20

**Authors:** Christoph Wiedemann, Kingsley Benjamin Obika, Sandra Liebscher, Jan Jirschitzka, Oliver Ohlenschlãger, Frank Bordusa

**Affiliations:** 1 Charles Tanford Protein Centre, Institute of Biochemistry and Biotechnology, Martin Luther University Halle-Wittenberg, Kurt-Mothes-Str. 3a, 06120 Halle, Germany; 2 Department of Chemistry, Institute of Biochemistry, University of Cologne, Zülpicher Str. 47, 50674 Cologne, Germany; 3 Leibniz Institute on Aging - Fritz Lipmann Institute, Beutenbergstr. 11, 07745 Jena, Germany; 4 Present Address: Faculty of Chemistry and Earth Sciences, Institute of Organic Chemistry and Macromolecular Chemistry & Cluster of Excellence “Balance of the Microverse”, Biostructural Interactions, Friedrich Schiller University Jena, Humboldtstraße 10, 07743 Jena, Germany

**Keywords:** Nuclear magnetic resonance spectroscopy, NMR, Intrinsically disordered protein, IDP, Human protein, Resonance chemical shift assignment, Structural and functional, Uncharacterized human protein

## Abstract

Even though the human genome project showed that our DNA contains a mere 20,000 to 25,000 protein coding genes, an unexpectedly large number of these proteins remain functionally uncharacterized. A structural characterization of these “unknown” proteins may help to identify possible cellular tasks. We therefore used a combination of bioinformatics and nuclear magnetic resonance spectroscopy to structurally de-orphanize one of these gene products, the 108 amino acid human uncharacterized protein CXorf51A. Both our bioinformatics analysis as well as the $^1$H, $^{13}$C, $^{15}$N backbone and near-complete side-chain chemical shift assignments indicate that it is an intrinsically disordered protein.

## Biological context

Of the 156,460 protein entries in the Protein Data Bank (PDB), roughly 7700 distinct proteins or parts of proteins are of human origin (June 2021). In comparison, the UniProt Knowledgebase lists approximately 20,400 human protein sequences which are both unique and have been reviewed appropriately (Bateman et al. 2021). Obviously, there is a large knowledge gap between the number of human proteins and the available structural and hence also detailed functional data. This knowledge gap needs to be addressed urgently, not only in light of a desire to understand the role of each human protein, but also as an untapped source of novel drug targets and for future biomedical advances.

In addition to focused attempts by individual laboratories centered on single proteins, consortia using high-throughput approaches that combine multiple techniques and screen large protein libraries (e.g. RIKEN Structural Genomics/Proteomics Initiative, Midwest Center for Structural Genomics, Structural Genomics Consortium) have emerged as effective forerunners in trying to determine every (human) protein structure possible. Nonetheless, as the abovementioned discrepancy in numbers between PDB and UniProt show, to date a significant number of proteins have escaped in-depth characterization. This may be either because they are difficult to express or purify or because they are unstable. Since X-ray crystallography is still the main work-horse in high-throughput facilities, enhanced protein dynamics, large flexible regions or lack of clearly defined tertiary structure all hinder efficient structure determination.

However, to complicate matters further, the relative amount of intrinsically disordered regions increases substantially from viruses to bacteria and ultimately higher eukaryotes. In humans, it is estimated that approximately 50% of all proteins either are intrinsically disordered (> 30% of disordered residues) or contain segments longer than 30 consecutive disordered residues (van der Lee et al. 2014; Deiana et al. 2019). Such intrinsically disordered proteins (IDPs) play important roles [in e.g. molecular recognition and assembly (e.g. Cdk inhibitor p21), protein modification (e.g. tumor suppressor p53) and entropic chain activities (e.g. RPA70 or MAP2)], but are particularly challenging to address (Tompa 2012; Kosol et al. 2013; van der Lee et al. 2014; Goretzki et al. 2021).

Nevertheless, a thorough structural and dynamic investigation is pivotal for our understanding of a protein’s molecular function and interaction. The recently released AlphaFold Database for protein structure prediction has the potential to accelerate our understanding at least of proteins with structural and dynamic homogeneity (Jumper et al. 2021). Among a range of spectroscopic techniques, and in contrast to X-ray crystallography or cryo-electron microscopy, nuclear magnetic resonance (NMR) spectroscopy provides information at atomic resolution on proteins displaying structural and dynamic heterogeneity.

Using a bioinformatics approach, we specifically searched the human reference proteome (UniProt proteome ID: UP00000564) for sequences of proteins that (i) had not yet been structurally characterized at an atomic level and (ii) would present feasible targets for standard liquid-state (NMR) spectroscopy due to their size, amino acid composition, known post-translational modifications, subcellular location and probability of soluble heterologous expression in *Escherichia coli*. A protein fullfilling all these criteria is the human CX05A.

The human protein CX05A (UniProt ID: A0A1B0GTR3) is 108 amino acids long with a high content of the basic residues lysine and arginine (19% and 11%, respectively) as well as serine (10%) and threonine (9%). The Human Genome Organization Gene Nomenclature Commitee provides the official full name: chromosome X open reading frame 51 A. The protein is also known as CXorf51 or CXorf51B and the chromosomal location of the coding sequence is Xq27.3. The human Protein Atlas [Uhlen et al. (2015), https://www.proteinatlas.org] lists relevant expression levels of human CX05A only for prostate and T-cells. There is no structural information of human CX05A or homologous proteins from other organisms available in the (PDB) and/or in the Biological Magnetic Resonance Bank (BMRB). In a very recent publication, the *CXorf51A* gene was identified in a transcriptome analysis as one of 52 genes that overlapped and were enriched (> eightfold) in the tunica media of the patent ductus arteriosus tissue compared to that of the closing ductus arteriosus tissues (Saito et al. 2021).

**Fig. 1:**
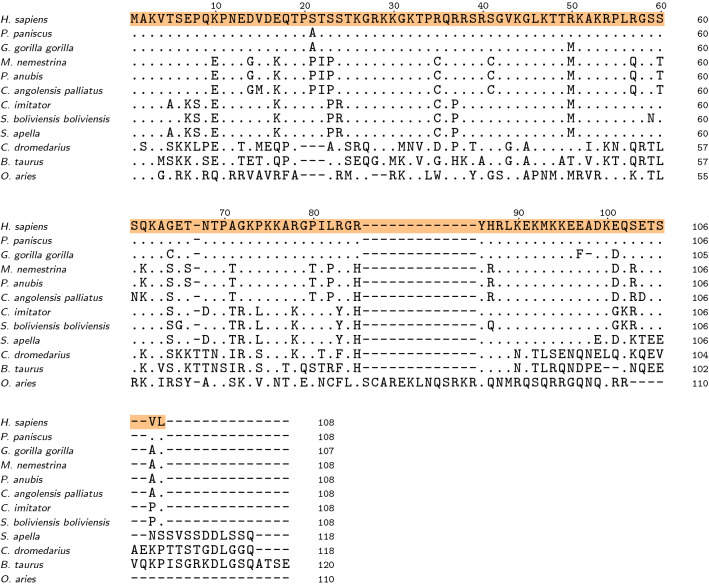
Sequence alignment [generated by Clustal Omega (Madeira et al. 2019)] of human uncharacterized protein CXorf51A and similar protein sequences found by a BLAST search in other selected species. The human CX05A sequence is used as consensus and highlighted in orange. Dots represent identical residues in other sequences and mismatches are shown in capital letters. Dashes indicate missing residues. Hominidae are *H. sapiens* (Human, UniProtKB: A0A1B0GTR3), *P. paniscus* (Bonobo, UniProtKB: A0A2R9B492) and *G. gorilla gorilla* (Western lowland gorilla, UniProtKB: A0A2I2Z775). *M. nemestrina* (Southern pig-tailed macaque, UniProtKB: A0A2K6CJ04), *P. anubis* (Olive baboon, UniProtKB: A0A2I3MM50) and *C. angolensis palliatus* (Angola colobus, UniProtKB: A0A2K5K7D9) are Old World monkeys. New World monkeys are *C. imitator* (Panamanian white-faced capuchin, UniProtKB: A0A2K5QKC3), *S. boliviensis boliviensis* (Black-capped squirrel monkey, UniProtKB: A0A2K6U040) and *S. apella* (Tufted capuchin, UniProtKB: A0A6J3FNG5). Additionally, a BLAST search reveals that CX05A-like proteins are also found in other Eutheria [e.g. *C. dromedarius* (Dromedary, UniProtKB: A0A5N4C141), *B. taurus* (Bovine, UniProtKB: G3MWQ4) and *O. aries* (Sheep, UniProtKB: O97965)]. Extending our search beyond the Eutheria clade revealed either a complete lack of this protein or a high degree of sequential dissimiliarity

We used the Basic Local Alignment Search Tool (BLAST) implemented in UniProt to find regions of local similarity between the human CX05A and protein sequences from other species (Fig. 1). Protein sequences producing significant alignments were only obtained from the clade of Eutheria. The BLAST search revealed a high sequence identity between the human CX05A and uncharacterized proteins from other Hominidae (e.g. *P. paniscus* and *G. gorilla gorilla* 99% and 94% identity, respectively). Proteins from Old and New World monkeys show sequence identities to the human CX05A of approximately 78%. The sequence identity between human CX05A and proteins from other Eutheria drop to 30% to 50%. Despite the lack of scientific knowledge on the CX05A protein, it seems to be of relevance.

Intriguingly, the fact that CX05A seems to be present only in Eutheria where it features a high degree of sequence identity, in particular amongst primates (Fig. 1), indicates an important and conserved structure and functional role for this protein in these animals.

In order to de-orphanize structural and functional uncharacterized human proteins, we report the nearly complete backbone and side-chain chemical shift assignments of CX05A and provide a chemical-shift-based secondary structure prediction.

## Methods and experiments

### Protein expression and purification

We obtained the full-length human *CX05A* gene, codon optimized for expression in *E. coli*, from Thermo Fischer Scientific (Germany). The gene was subcloned into a pET28a expression vector using NdeI and XhoI restriction enzymes, thereby introducing an N-terminal His$_6$-tag. We confirmed the subcloning process by DNA sequencing (LGC Genomics GmbH, Germany).

*Escherichia coli* BL21(DE3) cells were transformed with the resulting plasmid and plated onto kanamycin plates. A single colony from the plate was picked and grown in LB-Medium (supplemented with 50 μg/ml kanamycin) at 37 °C until an OD$_{600nm}$ 0.6. Cells were pelleted at 5250×*g* for 20 min using a Beckman Coulter SX4750A swinging bucket rotor, subsequently washed with 20 ml phosphate buffered saline and pelleted again. After resuspension in 250 ml M9 mineral salts medium (Azatian et al. 2019) supplemented with 1 g/l $^{15}$NH_4_Cl and 4 g/l $^{13}$C6-labeled glucose, gene expression was induced by adding 1 mM IPTG (isopropyl-1-$\beta$-d-galactopyranoside) and cells were grown at 37 °C. After 3 h the *E. coli* cells were harvested, immediately resuspended in 20 mM Na$_2$HPO$_4$, pH 7, 500 mM NaCl, 10mM imidazole, lysed by sonification and then centrifuged at 10,000×*g* for 40 min. The clear supernatant was applied to Ni-NTA affinity chromatography. The resin was washed with at least 10 column volumes each of 10 mM and 20 mM imidazole containing lysis buffer. Purified human CX05A was eluted with 0.25 M imidazole and subsequently further purified on a 16/60 HiLoad S75 size exclusion chromatography column (GE Healthcare) using a buffer containing 10 mM Na$_2$HPO$_4$, pH 6.5, 150 mM NaCl and finally dialyzed using a Slide-A-Lyzer TM dialysis cassette (10 kDa molecular weight cut off). The fractions containing human CX05A were pooled and concentrated. The purity of the obtained protein was confirmed by SDS-PAGE and mass spectrometry. The final concentration of the human CX05A NMR sample was about 400 μM.

Of note, the used construct has a thrombin cleavage site between the N-terminal His$_6$ tag and the native human CX05A sequence. Although no further thrombin cleavage site is predicted within human CX05A sequence, the addition of thrombin led to the rapid degradation of the protein. Therefore, the removal of the purification tag was omitted and the amino acid numbering is as follows: −19 to 0 indicates the purification tag, the native human CX05A sequence starts with methionine number 1.

### NMR spectroscopy

All NMR experiments on human CX05A were recorded at 283.2 K on a 700.5 MHz Bruker Avance III NMR spectrometer system equipped with a 5 mm TXI triple resonance probe (Bruker Biospin GmbH, Rheinstetten, Germany). The Topspin 3.6.2 software provided by Bruker was used for data acquisition and processing. 3-(trimethylsilyl)propane-1-sulfonate (DSS) at a final concentration of 0.1 mM was used for direct $^1$H chemical shift referencing as 0.00 ppm. $^{13}$C and $^{15}$N chemical shifts were referenced indirectly to the $^1$H DSS standard by the magnetogyric ratio (Wishart et al. 1995).

We assigned the backbone and side chain chemical shift resonances from a set of standard two- and three-dimensional heteronuclear experiments: [$^1$H,$^{15}$N]-HSQC, aliphatic and aromatic constant-time [$^1$H,$^{13}$C]-HSQC, HNCO (Kay et al. 1990; Ikura et al. 1990), HN(CA)CO (Clubb et al. 1992), HNCA (Kay et al. 1990; Farmer et al. 1992; Grzesiek and Bax 1992c), HN(CO)CA (Grzesiek and Bax 1992c; Bax and Ikura 1991), HNCACB (Grzesiek and Bax 1992a; Wittekind and Mueller 1993), HN(CO)CACB (Grzesiek and Bax 1992b), CC(CO)NH (Grzesiek et al. 1993) and [$^1$H,$^{15}$N]-TOCSY-HSQC (Marion et al. 1989). All experiments were implemented in the Bruker Topspin pulse program library and used without further modification. Each three-dimensional experiment was recorded using an exponential weighted non-uniform sampling scheme in the indirect dimensions for a sparsity of 25%. Compressed sensing with an iteratively reweighted least squares algorithm was used for data reconstruction (Holland et al. 2011; Kazimierczuk and Orekhov 2011). We analyzed the spectra using CcpNmr Analysis 2.5 (Vranken et al. 2005) within the NMRbox virtual environment (Maciejewski et al. 2017).

### Structure prediction

**Fig. 2:**
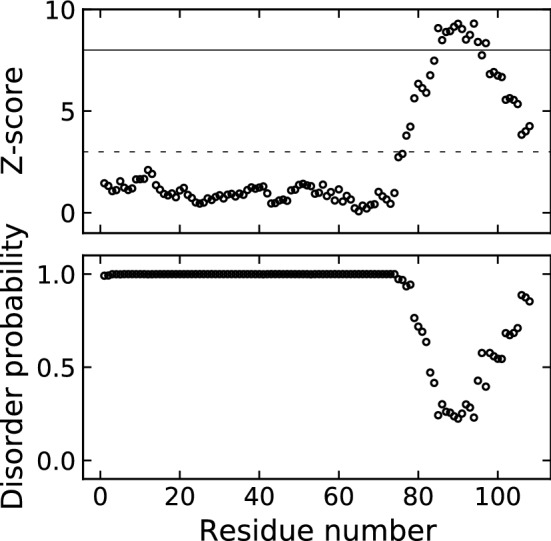
ODiNPred analysis of human CX05A predicts that the majority of the protein is disordered. The circles show the residue-specific Z-score (upper panel) and disorder probability (lower panel). A Z-score of 8.0 (solid line) is used to distinguish between disordered and ordered residues. A Z-score < 3.0 (dashed line) indicates fully disorder and values > 11.0 predict structured regions. A Z-score between > 3.0 and < 8.0 predicts the fractional formation of local order and 8.0 < Z < 11.0 corresponds to flexible loops next to ordered segments

We used the ODiNPred server (https://st-protein.chem.au.dk/odinpred) for the sequence-based prediction of structural disorder (Nielsen and Mulder 2019; Dass et al. 2020). The ODiNPred prediction is shown in Fig. 2 for the human CX05A. ODiNPred predicts human CX05A to be nearly completely intrinsically disordered. For a stretch of roughly 10 amino acids in the C-terminal part of CX05A the fractional formation of local order is predicted.

According to the predicted structural disorder, we used the POTENCI tool (https://st-protein02.chem.au.dk/potenci) to calculate the random coil chemical shifts for human CX05A based on the amino acid sequence taking into account temperature, pH value and ionic strength (Nielsen and Mulder 2018).

Additionally, we examined the secondary structure propensity (SSP) of CX05A based on the assigned backbone chemical shifts (Marsh et al. 2006).

## Extent of assignments and data deposition

We achieved the sequence specific resonance assignments for nearly all $^1$H, $^{13}$C and $^{15}$N spins of human CX05A using the suite of two- and three-dimensional NMR experiments mentioned in Methods and Experiments.

**Fig. 3:**
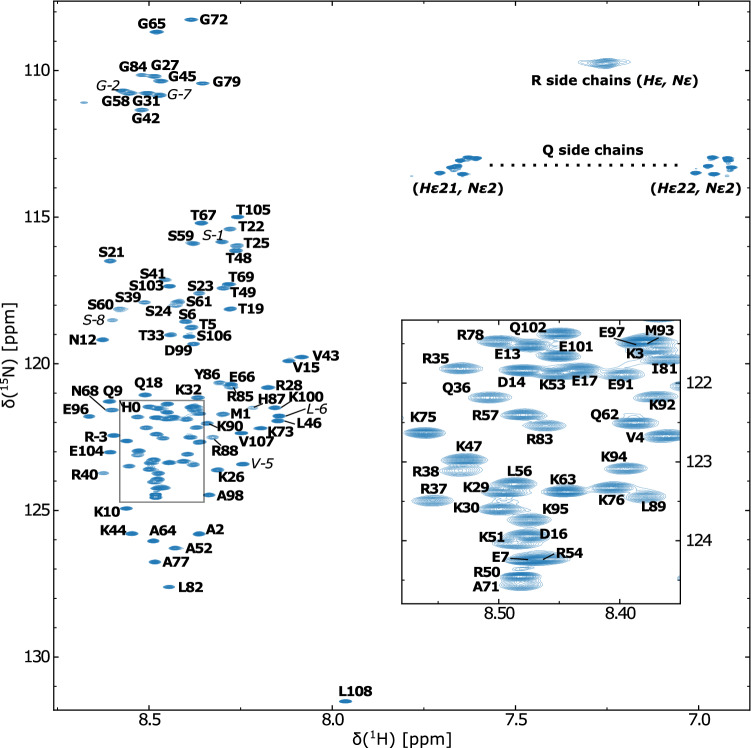
[$^1$H,$^{15}$N]-HSQC spectrum of $^{13}$C,$^{15}$N-labeled human CX05A in 10mM Na$_2$HPO$_4$, pH 6.5, 150 mM NaCl, 0.1 mM DSS, 90% H$_2$O/10% D$_2$O at 283.2 K. Assigned residues are annotated in bold face one letter amino acid code by the corresponding residue type and number. Residues originating from the N-terminal purification tag are marked in italic. Non-degenerate protons of the side chain amino groups are connected by a dashed line

In summary, the assignments of the backbone resonances $^1$H$^N$, $^1$H$^{\alpha }$, $^{13}$C$^{\prime }$ and $^{13}$C$^{\alpha }$) were completed to 100%. The assignment of $^{15}$N$^{\prime }$ was completed to 93%. Table 1 summarizes the extent of assignment and Fig. 3 shows the assigned [$^1$H,$^{15}$N]-HSQC spectrum of human CX05A.

**Table 1:** Extent of backbone and side chain assignment of human CX05A

Nucleus	Assigned (%)	Total number
$^1$H$^N$	100	100 out of 100^a^
$^{15}$N$'$	93	100 out of 108
$^{13}$C$'$	100	108 out of 108
$^1$H$^\alpha$	100	117 out of 117
$^1$H$^\beta$	100	177 out of 177
$^1$H$^\gamma$	100	136 out of 136
$^1$H$^\delta$	95	90 out of 95
$^1$H$^\epsilon$	66	42 out of 64
$^{13}$C$^{\alpha }$	100	108 out of 108
$^{13}$C$^{\beta }$	100	99 out of 99
$^{13}$C$^{\gamma }$	92	80 out of 87
$^{13}$C$^{\delta }$	77	51 out of 66
$^{13}$C$^{\epsilon }$	100	23 out of 23

^a^8 out of the 108 residues in human CX05A are prolines

**Fig. 4:**
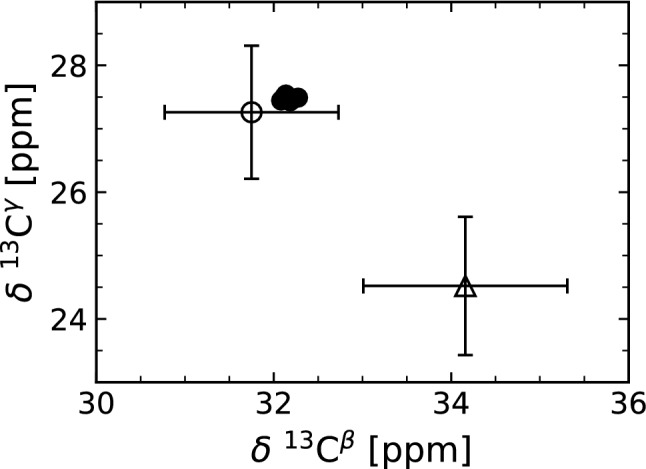
Proline $^{13}$C$^{\beta }$ and $^{13}$C$^{\gamma }$ chemical shift analysis for human CX05A. Filled circles correspond to the assigned proline $^{13}$C$^{\beta }$ and $^{13}$C$^{\gamma }$ chemical shifts. The open circle and the open triangle indicate the location of the mean (standard deviation shown as error bars) for a proline in *trans* and *cis* conformation, respectively (Schubert et al. 2002; Shen and Bax 2010)

We assigned the $^{13}$C$^{\beta }$ and $^{13}$C$^{\gamma }$ resonances for all 8 proline residues. The $^{13}$C$^{\beta }$ and $^{13}$C$^{\gamma }$ chemical shifts of all proline residues are in the range of 32.15 ± 0.05 ppm and 27.48 ± 0.03 ppm, respectively. Figure 4 shows a plot of the obtained $^{13}$C$^{\beta }$ versus $^{13}$C$^{\gamma }$ chemical shift values for all prolines in human CX05A. The mean difference of the proline $^{13}$C$^{\beta }$ and $^{13}$C$^{\gamma }$ chemical shifts is 4.67 ± 0.06 ppm. Consequently, we assume that for human CX05A in its major conformation all proline residues are in a *trans* Xaa-proline peptide bond (Schubert et al. 2002; Shen and Bax 2010). Nevertheless, this does not preclude the presence of minor populated states with prolines in *cis* configuration.

The signal dispersion in the $^1$H$^N$ dimension is reduced to the region between 8.1 and 8.6 ppm (Fig. 3). This limited $^1$H$^N$ chemical shift range is typically observed for proteins or protein parts without a well-defined three-dimensional structure. Therefore, the fast inter-conversion between different conformers or transient states results in ensemble-averaged random coil chemical shift values.

**Fig. 5:**
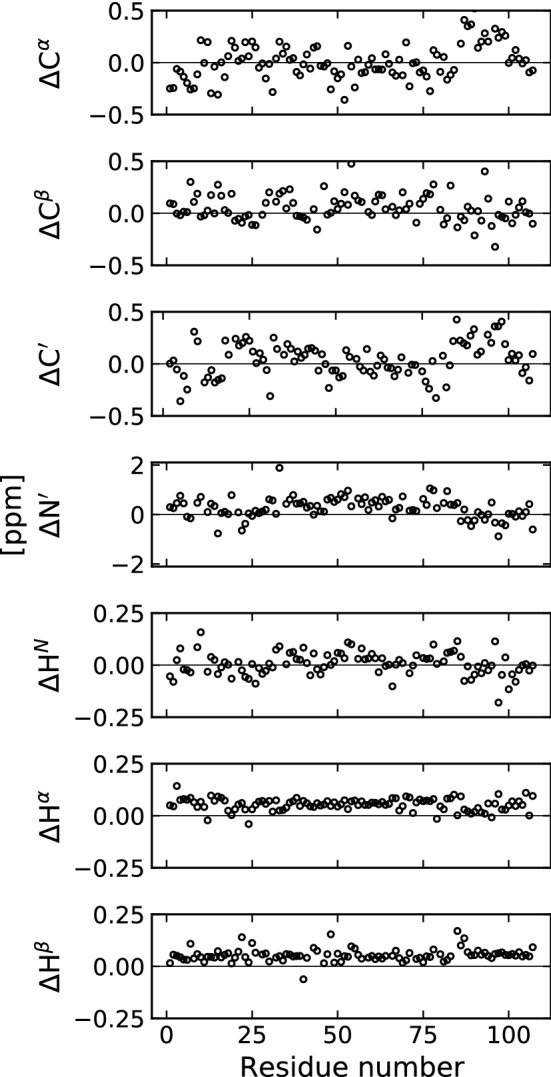
The difference between the experimentally obtained and the sequence-based predicted chemical shifts (using POTENCI) of CX05A is shown. For the prediction we took into account the NMR sample conditions (temperature, pH value and ionic strength)

To validate our obtained CX05A backbone and side chain assignments, we compared the assigned chemical shift values with the sequence based random coil chemical shifts for IDPs predicted from the POTENCI web server. The difference between the experimentally obtained and the predicted chemical shift values is shown in Fig. 5.

It is remarkable how well the measured and predicted chemcial shift values agree. Therefore, POTENCI is a valuable tool in the course of assigning chemical shifts of IDPs. The mean differences between the measured and the POTENCI-predicted values are as follow: $^{13}$C$^\alpha$ 0.017 ± 0.189 ppm, $^{13}$C$^{\beta }$ 0.052 ± 0.128 ppm, $^{13}$C$'$ 0.042 ±0.166 ppm, $^{15}$N$'$ 0.267 ± 0.423 ppm, $^1$H$^N$ 0.007 ± 0.056 ppm, $^1$H$^\alpha$ 0.054 ± 0.029 ppm and $^1$H$^\beta$ 0.054 ± 0.030 ppm.

**Fig. 6:**
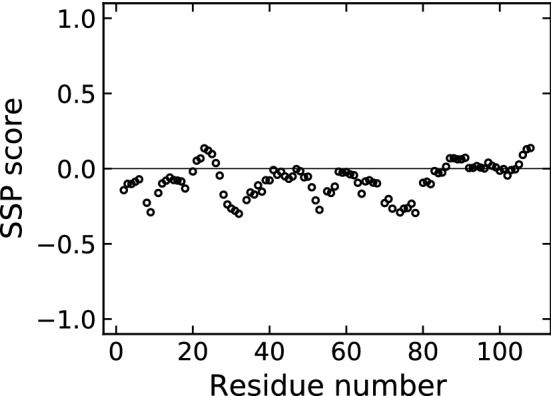
The sequence specific secondary structure propensity (SSP) scores are depicted. Values below and above 0 indicate $\beta$-sheet and helical-structure propensity, respectively. A SSP score of 1 reflects fully formed helical-structure. Fully formed $\beta$–structure is indicated by a SSP value of -1. As recommended for disordered proteins, only $^{13}$C$^{\alpha }$, $^{13}$C$^{\beta }$ and $^1$H$^\alpha$ chemical shifts were applied and residues immediately preceding prolines were considered when running the SSP script

The amino acid sequence based disorder prediction using ODiNPred identifies human CX05A as almost entire intrinsically disordered (Fig. 2). In agreement with the [$^1$H,$^{15}$N]-HSQC spectrum (Fig. 3), the CSI 3.0 web server (Hafsa et al. 2015) predicts an all-coil conformation for CX05A based on our chemical shift assignments (data not shown). Likewise, the secondary structure propensity method (Marsh et al. 2006) used to reveal potential structural elements (based on the chemical shift data) could not detect significant secondary structure content (1.2% and 9.5%, respectively). Figure 6 shows the sequence specific secondary structure propensity. This supports the observation made from the [$^1$H,$^{15}$N]-HSQC spectrum (Fig. 3) and the sequence based disorder prediction.

Together, the experimental data and the structural predictions clearly show that human CX05A is an IDP under buffer conditions chosen to somewhat mimick cellular conditions while providing optimal conditions for NMR spectroscopy. Interestingly, the C-terminal region of the protein may have fractional local order. However, if the C-terminal part is a molecular recognition feature that might fold upon binding is still speculative. In addition, post-translational modifications may further change the structural dynamics of this protein. It is likely that in a cellular context certain serines, threonines or the tyrosine are phosphorylation sites. At least several potential consensus motifs for different kinases are present in CX05A (e.g. Calmodulin-Dependent Protein Kinase II [R-X-X-S/T]: S41, S60; Protein Kinase A [R/K-X-S/T]: S39, S59; Serine/threonine-protein kinase Nek6 [L-X-X-S/T]: T49). The regulation of the function of CX05A by de/phosphorylation thus seems to be possible. Although interaction partners and function of CX05A are still unclear, the high content of basic amino acids and frequent basic residue patches (e.g. RG, RS, RR, KG, KK, KR) foster the speculation of a DNA/RNA-binding protein.

Using a straightforward approach, we identified the structurally “unknown” human protein CX05A as a suitable candidate for NMR spectroscopy and subsequent structural characterization. Hopefully, similar endeavors by us and others will aid to reduce the current structural knowledge gap one challenging protein at a time.

## Data Availability

The assigned $^1$H, $^{13}$C and $^{15}$N chemical shift values of the human CX05A are available in the BMRB (https://bmrb.io) under the Accession No. 50944.
